# Effect of platelet concentrates for pain and symptom management in oral lichen planus: an evidence-based systematic review

**DOI:** 10.1186/s12903-023-03296-1

**Published:** 2023-08-25

**Authors:** Yuanmei Zhang, Chenhao Mao, Juanfang Zhu, Weiwei Yu, Zhejun Wang, Yanli Wang, Quanlong Kan

**Affiliations:** 1 The Department of Prosthodontics, The First Affiliated Hospital of Zhengzhou University, Zhengzhou, China; 2 Henan University of Economics and Law, Huang He Business School, Zhengzhou, China; 3 The Department of Orthodontics, The First Affiliated Hospital of Zhengzhou University, Zhengzhou, China; 4 Wuhan University, The State Key Laboratory Breeding Base of Basic Science of Stomatology (Hubei- MOST) & Key Laboratory of Oral Biomedicine Ministry of Education, School & Hospital of Stomatology Wuhan, Hubei, CN China; 5 The Department of Interventional Radiography, The First Affiliated Hospital of Zhengzhou University, Zhengzhou, China

**Keywords:** Oral lichen planus, Platelet concentrates, Steroid, Topical treatment

## Abstract

**Background:**

Platelet Concentrate (PC) injection therapy has shown potential as a local therapy for oral lichen planus (OLP). However, its safety and efficacy have not yet been fully established. Our research compared the efficacy of PC with topical steroid treatment in alleviating pain and symptoms related to OLP. We aims to present evidence-based alternatives that dentists can use to improve patient outcomes while reducing potential side effects.

**Methods:**

We conducted a systematic search of five electronic databases up to April 2023, including Embase, Cochrane Central Register of Controlled Trials, PubMed, OVID Medline, and WanFang, to evaluate PCs' efficacy compared to topical corticosteroid therapy for OLP. The literature quality was assessed using the Cochrane ROB tool. A fixed-effects model was used to determine the Weighted Mean Difference (WMD) and Mean Difference (MD) at a 95% confidence interval (CI) for pain severity and other relevant clinical indicators.

**Results:**

The comparison between topical corticosteroid therapy and PCs showed no significant difference for pain relief (WMD = -0.07, CI = 95% -0.34 to 0.19), symptom improvement (MD = -0.21, CI = 95% -0.55 to 0.13), or the severity of included lesions measured by REU scores (MD = -0.25, CI = 95% -0.32 to 0.82).

**Conclusions:**

Locally injected PC have been found efficient in managing oral lichen planus, indicating that they are a promising alternative option to steroid therapy for OLP patients, particularly those who have not responded favorably to steroid therapy. However, further research is needed to establish determining the recurrence rate and long-term adverse effects.

**Trial registration:**

The systematic review protocol has been registered in advance with the PROSPERO database (CRD42023415372).

**Supplementary Information:**

The online version contains supplementary material available at 10.1186/s12903-023-03296-1.

## Background

Oral Lichen Planus (OLP) is among the most prevalent dermatologic diseases that occurs within the oral cavity, affecting approximately 0.5% to 2% of the global population [1]. It is characterized by white reticular or erosive lesions on the oral mucosa, which can cause pain, discomfort and impaired daily activities for many patients [2, 3]. The condition mainly affects women over the age of 40 with a 2:1 female-to-male ratio. Although several therapeutic options are available for managing OLP, including corticosteroids and calcineurin inhibitors, photochemical therapy, and retinoids; topical corticosteroids are commonly used as the primary pharmacological remedy, and intralesional corticosteroids are also effective in managing OLP by enabling high drug concentrations at the injected site [4, 5].While numerous treatments are accessible for the disease, they usually cause side effects and do not ensure a permanent cure. For example, extended use of intralesional corticosteroids has been linked to several systemic adverse effects, including taste loss, mouth dryness, candidal infection, mucosal atrophy and so on.

Platelet concentrates (PCs) are autogenous substances obtained from blood, which contain supraphysiological levels of platelets and growth factors(GFs). Autologous biological product derived from the patient's blood, are widely used in regenerative medicine due to their autogenous sources of GFs that can induce tissue repair and regeneration, while avoiding any potential immunological or allergic reactions [6]. PCs are obtained through blood centrifugation, resulting in the concentration of GFs and cytokines that exert a beneficial effect on inflammation, angiogenesis, stem cell migration and proliferation, which in turn enhancing the potential for repair and regeneration [7, 8]. Platelet-rich plasma (PRP), plasma rich in growth factors, Injectable-platelet-rich fibrin (i-PRF), and concentrated growth factors are examples of such products, classified according to their preparation protocols [9].In recent research, Dohle et al. demonstrated that PRF has a positive impact on wound healing and angiogenesis, while Fujioka-Kobayashi showed that dense granules within platelets release mediators like histamine, serotonin, and dopamine that aid in pain reduction when included in platelet concentrates [10, 11]. The efficacy of platelet concentrates in treating OLP patients by reducing the immune response and alleviating symptoms has been supported by several clinical trials [12–15].

As PCs products with fewer or no side effects become increasingly popular, it is crucial to compare their clinical performance with that of topical corticosteroids, especially since the most significant expectation among OLP patients from their medications is rapid pain relief [16]. Therefore, this is the first research evaluates the effectiveness of blood-derived products compared to steroid therapy for OLP treatment. By providing evidence-based recommendations, this meta-analysis supports clinicians in choosing suitable therapies, particularly in cases where systemic diseases prohibit the use of steroids or to avoid their side effects.

## Methods

The protocol was also registered in advance with the PROSPERO database (CRD42023415372).

### Database and search strategy

The studies included were met the following inclusion criteria (without language restriction):

- P (population): Adult patients who were clinically and/or histologically diagnosed as OLP accordance to WHO criteria without history of corticosteroid therapy in topical lesion (in the oral cavity) in the past 2 weeks or systemic delivery in the past 4 weeks.
- I(Intervention): Patients were treated with injections of PC derivatives, such as PRP and i-PRF, throughout the course of treatment [17].
- C (Comparison): The interventions in this study comprised receiving corticosteroid injection therapy over the course of treatment. The outcome measures for this study comprised pain relief, assessed through changes in the 0–10 scale, using visual analog scale (VAS) or numerical rating scale (NRS). Clinical resolution by Thongprasom (Sign score) was also evaluated using experimental and control procedures. Lesion severity on each site was scored based on Reticulation/keratosis, erythema, and ulceration (REU) score and lesion size, while reported side effects were also recorded.
- S (study): Controlled trials, randomized controlled trials, randomized cross-over design trials and cohort studies.

A comprehensive search was performed in databases, including Embase, Cochrane Central Register of Controlled Trials, PubMed, OVID Medline, and WanFang, were searched from their inception up to April 2023 (see the appendix in the electronic Supplementary Table S1). Two reviewers (YM and CH) assessed all titles and abstracts, and literature management was conducted using Endnote. Potentially eligible abstracts and abstracts with disagreement or insufficient information were evaluated in full-text screening.

### Data extraction

Two reviewers (CH and JF) independently extracted data from relevant research papers. The collected information comprised authors, country, year of publication, study design, number of subjects, haracteristics of the study population such as age and gender, experiment and control groups, evaluation methods, adverse reactions, and main study findings. In case of any discrepancies, they were resolved through discussion and consensus. Whenever required, the authors were contacted for additional data.

### Quality assessment

The methodological quality of RCTs included in our research will undergo independent evaluation by two reviewers(YM and WW). In cases of disagreement, they will resolve by a third reviewer's judgment. To assess the quality of the involved citations, we use the Cochrane ROB tool [18]. The following items will be evaluated: (1) Randomization process, (2) Deviations from intended interventions, (3) Missing outcome data, (4) Measurement of the outcome, (5) Selection of the reported result, (6) Overall.

### Statistical analysis

The WMD for VAS or NRS scores, as well as the MD for sign scores and REU were analyzed for both experiment and control procedures after therapy. Descriptive and statistical analyses were conducted, with an evaluation of heterogeneity through Q statistic and inconsistency index (I2) statistic. The fixed-effects model was used when heterogeneity was present (I2 > 50%), while a random-effects model was utilized for data without significant heterogeneity (I2 < 50%). Subgroup analysis was performed by different blood derivative components. A sensitivity analysis was performed, sequentially eliminating each study to verify the stability of results. All analyses were conducted using To evaluate potential publication bias, both Beggs' and Eggers' tests were carried out with a statistical significance level set at α = 0.05.All statistical analyses were performed using STATA software (version 15.1, Stata/SE).

## Results

### Search results

**Fig. 1:**
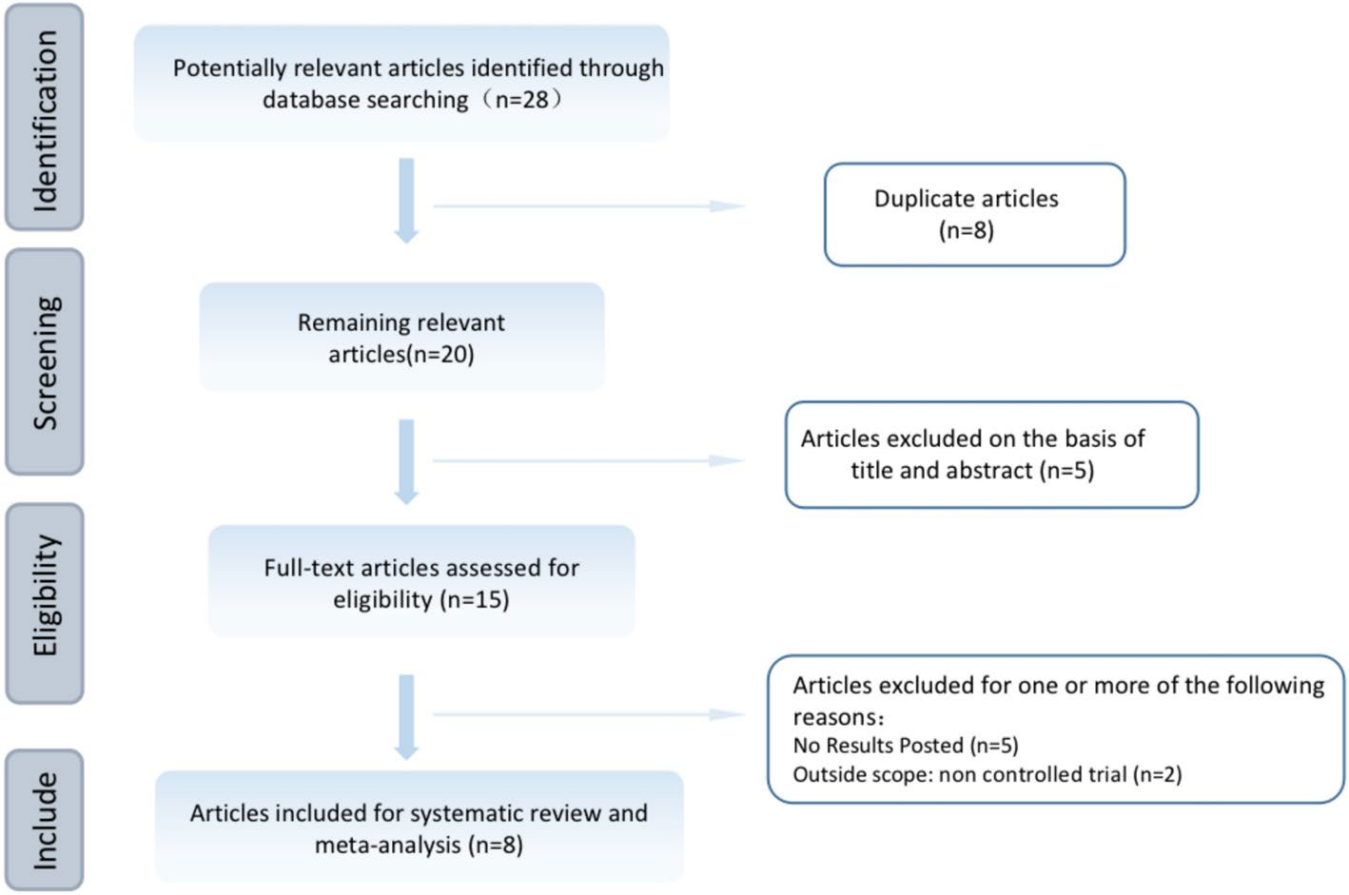
Flowchart of study selection

According to our initial database search, we initially identified 81 records. After eliminating duplicates, we screened 29 records. Following a thorough review of titles and abstracts, we excluded 15 records, leaving us with 8 full-text articles for further analysis. The detailed search flowchart is shown (see Fig. 1).

### Characteristics of included studies

The main information and characteristics of the selected trials are retrievable from their original publications and have been summarized (i.e. Table 1). Eight studies were included in our analysis, of which 1 was Non-randomized controlled trial while 7 were randomized controlled trials. All the studies compared the efficacy of blood derivatives to topical corticosteroid therapy in pain relief. Five of these studies investigated the efficacy of i-PRF compared to local corticosteroids, while the other three papers used PRP as the therapeutic agents. Triamcinolone (TA) was the most commonly used steroid in the treatment, except in one trial where methylprednisolone acetate was chosen. Clinical sign scores were reported in four studies as well as changes in lesion area recorded in REU and lesion size before and after the treatment. Other related indicators included recurrence rate percentage, effective rate, and Oral Health Impact Profile (OHIP-14); however, insufficient data was available for data analysis.

**Table 1:** Characteristics of the 8 studies included in this meta-analysis

			Sample Size					Intervention					
Author (year)	Study design	Country	Experiment group	Control group	Age range (mean)	Female/Male	Type of OLP	Experiment group	Control group	Evaluation methods	Follow-up period	Outcome	Adverse effect
ElGhareeb 2023 [19]	clinical trail	Egypt	12	12	49.55±11.67	14/10	Erosive or Reticular or Mixed	Intralesional PRP every two weeks	Intralesional TA every two weeks	VAS，REU，complete response rate	3m	Intralesional administration of PRP presents itself as a viable and secure modality for the treatment of OLP in patients.	There were no statistically significant differences between the studied groups in therapeutic response
Hijazi 2022 [20]	RCT	Egypt	10	10	46.45±10.37	18/2	erosive	Intralesional PRP every two weeks	Intralesional TA every two weeks	VAS，Sign score	3m	PRP injections could be considered as an effective alternative single treatment modality for EOLP.	-
Al‐Hallak 2021 [15]	RCT	Syria	12	12	48±12.7	9/3	Erosive or Reticular or Mixed	Intralesional i-PRF every two weeks	Intralesional TA every two weeks	VAS，REU，Percentage of OLP recurrence	3m	Intralesional injection with TA showed more effectiveness than i-PRF in the management of OPL lesions.	-
Saglam 2021 [14]	RCT	Turkey	24	24	24-76	14/10	erosive	Intralesional i-PRF every 15-day	Intralesional methylprednisolone acetate every 15-day	VAS，lesion size，OHIP-14	6m	In patients with EOLP, both methods decreased pain and lesion size similarly, and both increased satisfaction.	no systemic side effects
Bennardo 2021 [21]	RCT	Italy	9	9	59.56±3.57	6/3	Erosive or Reticular or Mixed	Intralesional i-PRF every week	Intralesional TA every week	VAS，lesion size	2m	i-PRF seems to be as effective as TA	no side effects were observed
LH Zheng 2021 [22]	RCT	China	19	19	32-81	25/13	Erosive or Reticular or Mixed	Intralesional i-PRF every week	Intralesional TA every week	VAS，Sign score, lesion size， effective rate, recurrence rate	3m	i-PRF seems to be as effective as TA.	the incidence of adverse reactions in i-PRF group is lower than that of TA group
Ahuja 2020 [23]	RCT	India	10	10	28-60	18/2	erosive	Intralesional PRP every week	Intralesional TA every week	VAS, erythema scores, Mean Lesion size	4m	Comparison of the pain reduction, size of lesion and erythema scores between the two groups, the difference was found to be statistically insignificant	no side effects were observed.
Tunalı 2018 [24]	RCT	Turkey	13	13	-	-	Erosive or Reticular or Mixed	Intralesional i-PRF	Intralesional corticosteroids	VAS, OHQoL, Sign	-	I-PRF injection may be effective in the symptomatic treatment of OLP	-

### Quality assessment

**Fig. 2:**
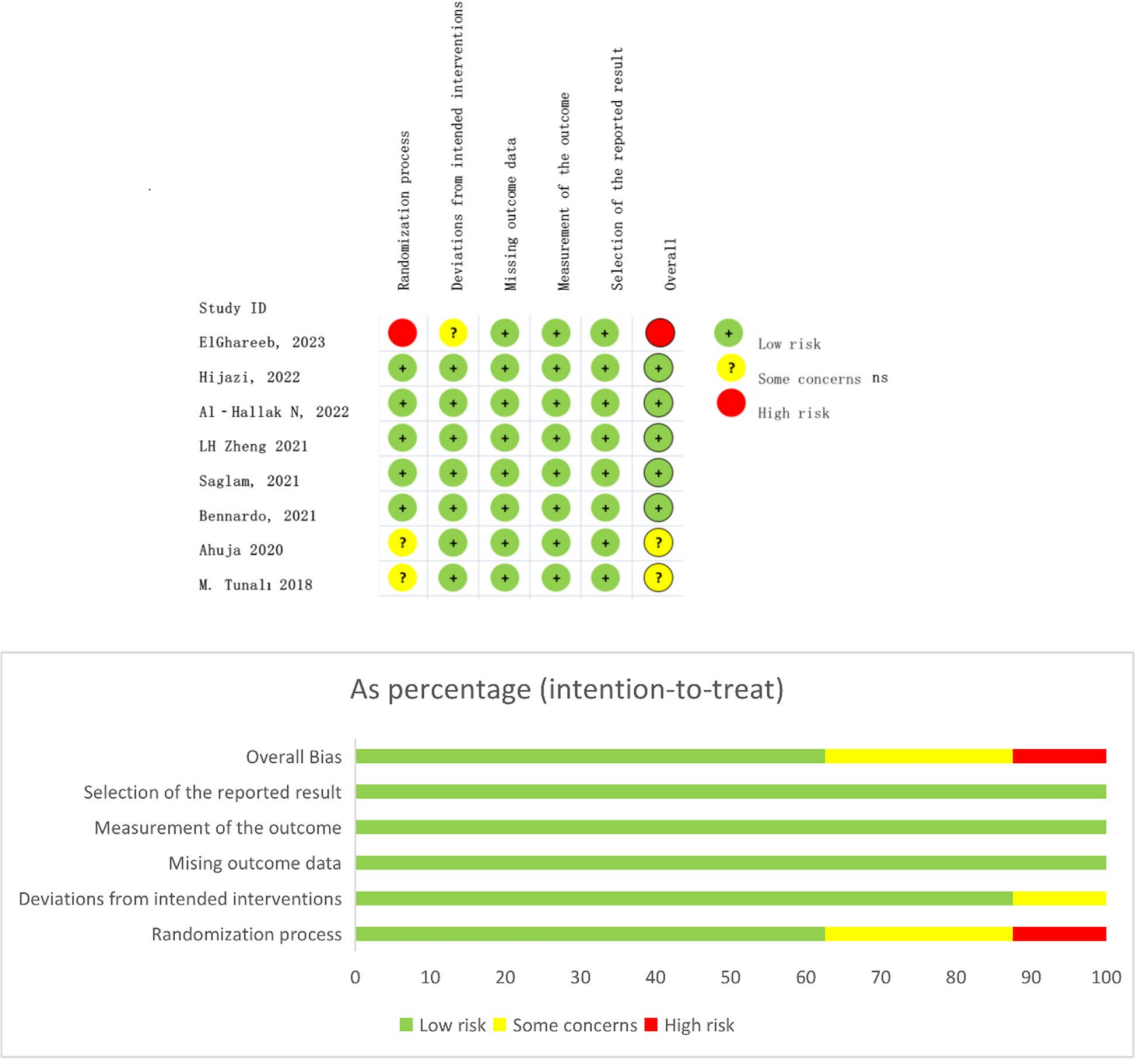
Risk of bias summary

The methodological quality of the nine RCTs was evaluated based on the Cochrane ROB tool and presented (see Fig. 2). This figure provides a summary of the risk of bias assessment for all studies included in our research. Due to the specificity of the treatment, blinding patients was not feasible since blood preparation for i-PRF or PRP was required. In addition, researchers could not be blinded due to the distinct color difference between the two drugs and the need to inform patients about potential adverse reactions before treatment. Although most study designs employed blinding techniques for outcome assessors, it was not possible to blind patients and researchers given these circumstances. As a result, the quality assessment of the study results may be downgraded due to this limitation.

It is worth noting that the clinical study without randomization was classified as high-risk, while one prospective randomized clinical trial was deemed of some concern due to its inherent limitations in describing its randomization process. The conference summary was also classified as moderate risk due to its lack of specific details. Conversely, all other included in studies were classified as low risk of bias. (see the appendix in the electronic Supplementary Table S2).

## Effects of interventions

### Pain score

**Fig. 3:**
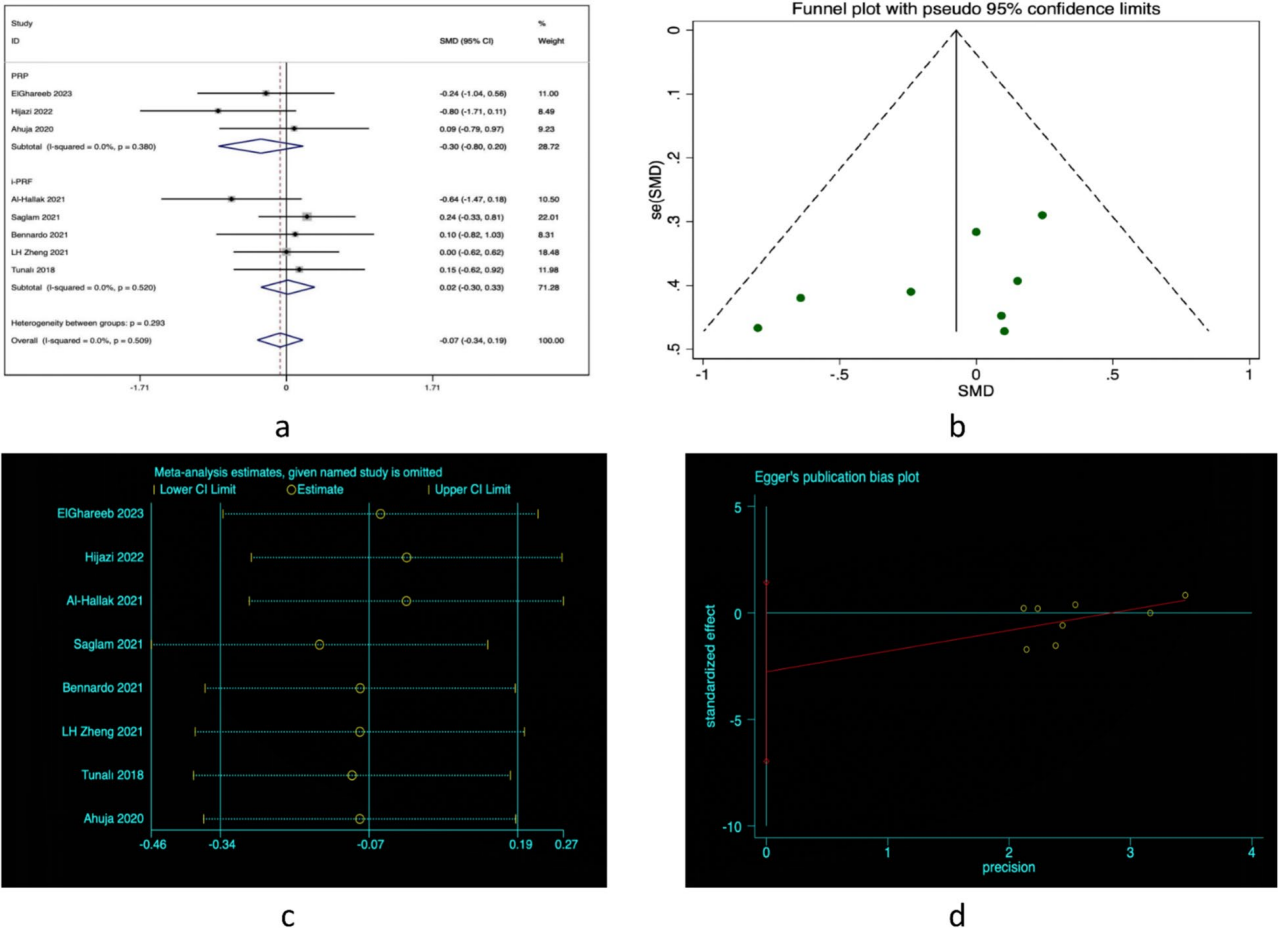
**a** Forest plot in pain relief. **b** Funnel plot. **c** Sensitivity analysis plot. **d** Egger’s funnel plot

Eight clinical trials [14, 15, 19–24] comparing the efficacy of PCs (i-PRF and PRP) to steroid treatment in reducing pain associated with OLP. The results of this analysis revealed no significant difference between the two plasma extracts, as the weighted mean difference (WMD) was (WMD = -0.07,CI = 95% -0.34 to 0.19) as shown(see Fig. 3a). This indicates that blood derivatives may serve as a viable alternative therapy for managing pain in individuals with OLP. Heterogeneity among the studies was minimal (I^2^ = 0%), as indicated by the test for heterogeneity. Although there is a potential for small sample size bias, the funnel plot displayed a symmetrical distribution. Sensitivity analysis confirmed the stability of the analysis. Subgroup analysis was conducted on the PRP group (WMD = -0.30,CI = 95%-0.8to 0.2) and the i-PRF group (WMD = 0.02,CI = 95%-0.30 to 0.33). Based on the results of our study, the begg's test showed a non-significant *p* value of 0.266, indicating no evidence of publication bias. Similarly, the egger's test also yielded a non-significant p value of 0.158, further supporting the absence of publication bias(see Fig. 3b,c,d).

### Sign score

**Fig. 4:**
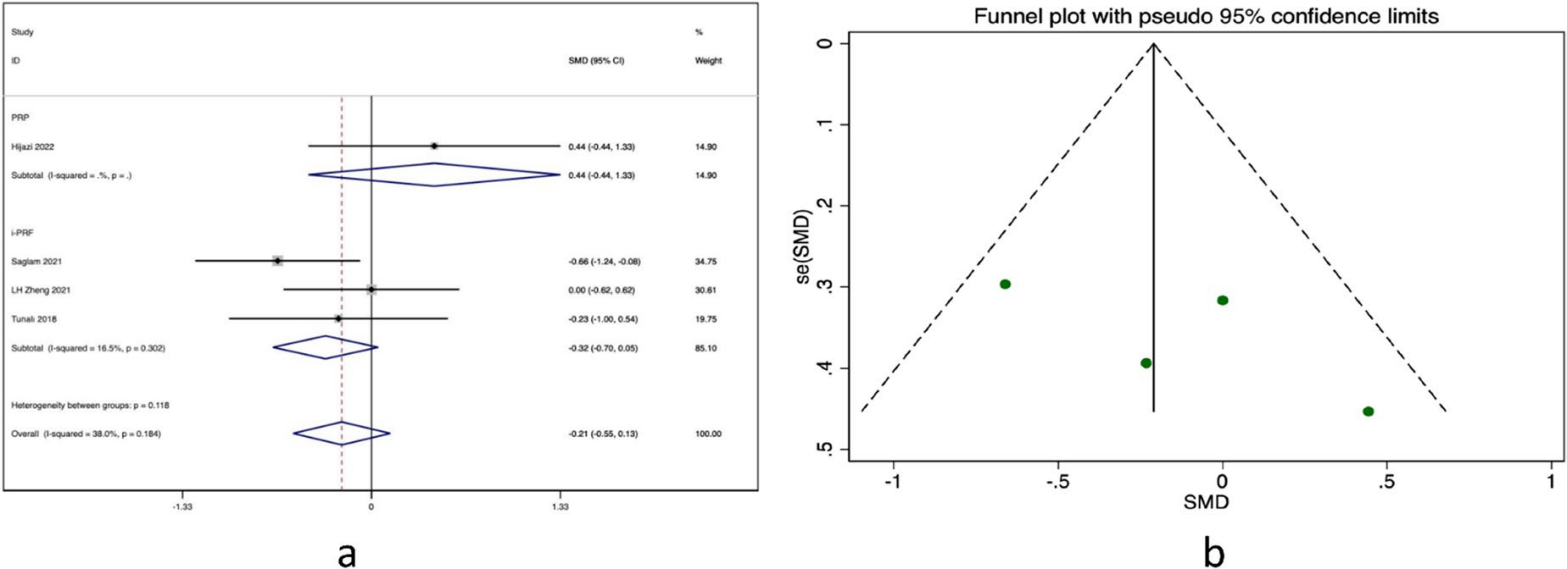
**a** Forest plot in Sign scores, **b** Funnel plot

Four clinical trials [14, 20, 22, 24] were conducted to compare the improvement of clinical symptoms between blood derivatives and steroid treatment groups. However, the mean difference between the two groups was found to be(MD = -0.21 95% CI: -0.55 to 0.13), indicating no significant difference as shown (see Fig. 4a). Although the funnel plot was symmetrical, a small sample size bias cannot be ruled out (see Fig. 4b). To obtain a more comprehensive understanding of the study outcomes, subgroup analysis was performed on the PRP group (MD = 0.44, 95% CI: -0.44 to 1.33) and i-PRF group (MD = -0.32, 95% CI: -0.70 to 0.05). The heterogeneity between the groups was low (I^2^ = 38.0%).

### REU score

**Fig. 5:**
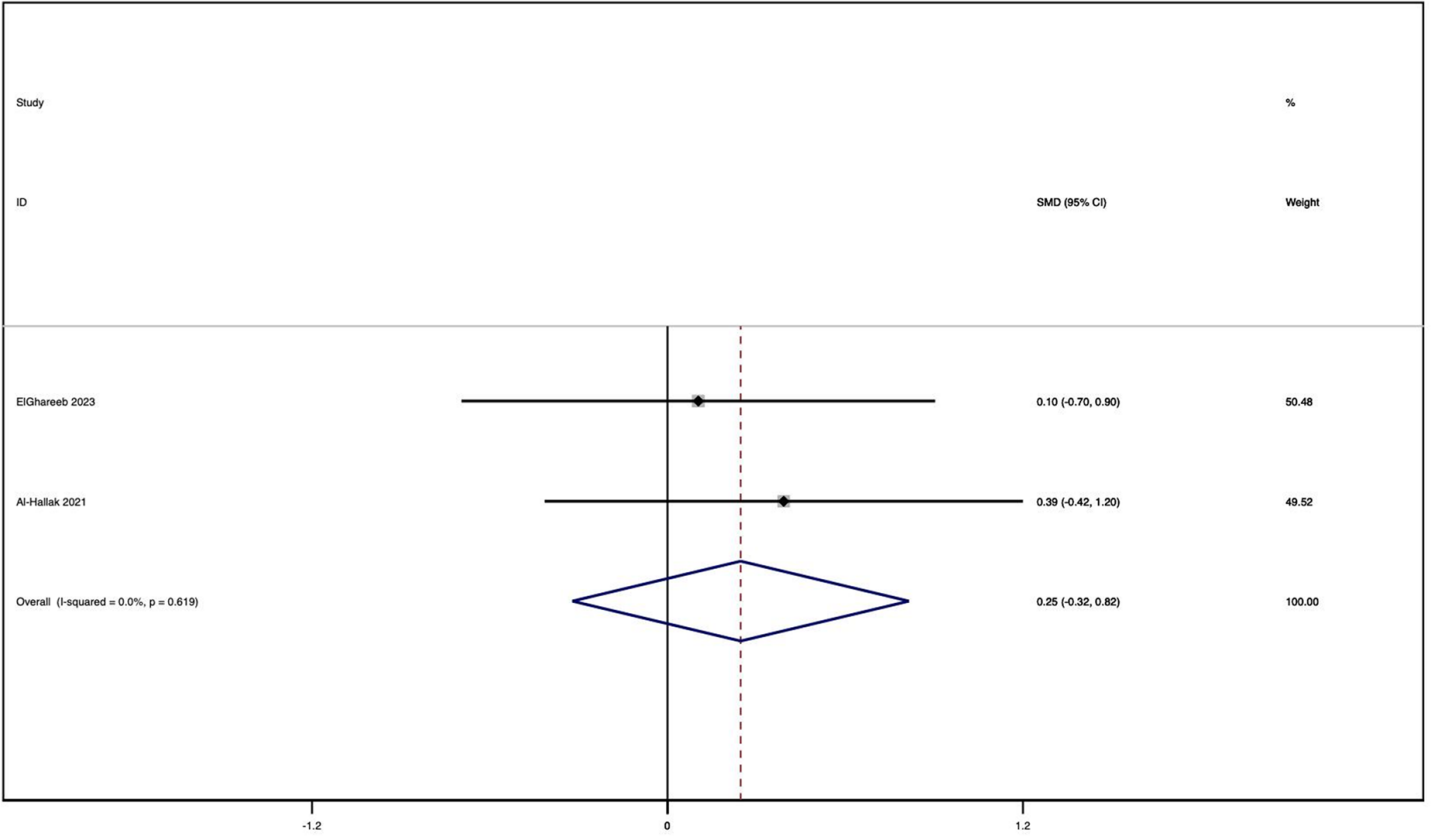
Forest plot in reticulation/keratosis, erythema, and ulceration score

Two clinical trials [15, 19] were conducted to evaluate the clinical features and severity of lesions using the REU score. The analysis of these trials indicated no significant difference between the two treatment groups, as the mean difference was found to be minimal (MD = 0.25,CI = 95% -0.32 to 0.82) shown(see Fig. 5). Also, the study revealed a substantial reduction in the clinical severity of both treatment groups.

### Lesion Size

Two studies [21, 22] were conducted to compare the effectiveness of i-PRF and TA treatments in healing mucosal and ulcerative tissue lesions. In the first study [21], both treatments were found to be effective when measured using a calibrated periodontal probe. Nevertheless, the TA group exhibited a slightly higher total effective rate compared to the i-PRF group, and the difference was not significant (P > 0.05). In the second study [22], both treatments were successful, but i-PRF resulted in a greater average modification of the affected area compared to TA through the use of Adobe Photoshop software.

### Side effect

In the included studies, 4 studies [14, 19, 21, 23] did not report any significant intraoperative or postoperative complications. One study [22] reported a candida infection within the TA group, and mucosal pain in one patient following medication administration. However, no statistical difference in the occurrence of adverse effects (*P* > 0.05). Another study [19] reported that patients who received PRP experienced a statistically significant increase in side effects, notably pain. Three studies [15, 20, 24] made no mention of any side effects.

### Other indices

In Al-Hallak's study [15], the recurrence rate was found to be 16.7% during the three-month treatment period. during the corresponding three-month follow-up, two patients demonstrated mild recurrence symptoms in the bilateral buccal mucosa. LH Zheng [22] reported that after a three-month follow-up observation, the recurrence rates for both groups of cases were 14.29% and 17.65%. The difference in rates was not statistically significant. Saglam's study [14] revealed that no significant difference in oral hygiene between two treatment groups, as was assessed using the the OHIP-14.In study by ElGhareeb [19], PRP resulted in a 66.6% complete response among patients with erosive OLP.

## Discussion

Oral lichen planus is an autoimmune, chronic inflammatory disorder which is identified by the basal layer of the oral epithelium with T-cell mediation [25]. While its exact cause remains unknown, growing evidence suggests that immune dysregulation significantly contributes to its development through multiple mechanisms. Platelets serve as potential sources releasing anti-inflammatory cytokines and regulating inflammatory mediators [26]. PCs contain a diverse array of GFs, including vascular endothelial growth factor, insulin-like growth factor-1, basih factor β-1 and platelet-derived growth factor-BB, which are released upon activation [27]. These GFs stimulate mesenchymal cell recruitment, regulate keratinocyte and regulatory T-cell functions, inhibit inflammatory cytokine and transcription factor expression, and reverse extracellular matrix destruction in OLP lesions [28]. PRP and i-PRF contain TGF-β, PDGF, EGF, VEGF, IGF [29], and fibronectin, boost cell proliferation, facilitate angiogenesis, and promote wound healing-related cell migration, all of which aid in tissue regeneration [30, 31]. Additionally, PRP and i-PRF release GFs and cytokines that significantly regenerate tissues through their angiogenesis properties [32]. The 3D fibrin matrix containing autologous plasma extract carries cytokines and GFs, both of which play vital roles in the regeneration process. Huber et al. found that PRP promotes anti-inflammatory cytokines production, which interact with soluble receptors and inhibitors to regulate inflammation and growth factor activity [33, 34]. Additionally, i-PRF actively augments proliferation and migration of endothelial cell and fibroblast, promoting tissue regeneration and wound healing by stimulating cell migration and proliferation during the proliferation phase [11].

This article summarizes the results of eight studies that assessed the efficacy of autologous blood derivatives, including PRP and i-PRF, as well as corticosteroid injections for managing OLP. Both blood derivatives and corticosteroids were found to be effective in relief pain and clinical scores in OLP patients. However, some discrepancies exist in earlier therapeutic responses. The Elghareeb [19] study reported a higher frequency of side effects, particularly pain, with PRP treatment compared to steroids. Conversely, the Al-Hallak study [15] showed a significant decrease in pain scores for both treatment groups, while the Ahuja study [23] revealed that PRP provided comparable or better comparative results than topical steroids in later phases of treatment, in spite of initially showed slightly less reduction in assessed parameters. Notably, PRP exhibited slightly less reduction in symptoms comparing with i-PRF during the first two weeks of treatment [35]. It appears that i-PRF may have a faster clinical response than PRP in managing OLP. Furthermore, the Hijazi study [20] suggested that although PRP had a slower clinical response than TA injections, both treatments exhibited similar complete remission rates, with no significant differences observed in pain score, Sign score, or lesion area remission. The observed differences could be attributed to variations in the duration of release and peak time of GFs reported across studies [36, 37].The UE Shinnawi study [38] also supports this notion, showing a clear improvement in symptoms with blood derivatives after the first two weeks of treatment. Several other studies have assessed the efficacy of injection PCs in managing refractory erosive OLP patients who showed no response to corticosteroid treatment [39]. The results revealed that PCs therapy is an effective approach for treating atrophic-erosive lesions associated with this condition, which is generally unresponsive to corticosteroids. In a study by Samiee et al. [40], involved ten female patients, and found that PCs treatment resulted in complete symptom absence within an average of 13 months after the procedure. Similarly, another study by Anitua et al. [41], which included four female participants, reported significant relief in pain scores and complete healing following one or two PCs infiltrations. Additionally, Piñas's study [13] study with fifteen participants demonstrated PCs therapy's ability to significantly reduce pain scores with a mean follow-up duration during which participants remained symptom-free of 47.16 months. Our findings provide substantial evidence of platelets derivates therapy's effectiveness as a treatment option for refractory erosive OLP.

Overall, no statistically significant difference were found in recurrence rates or side effects in the literature included in the study, supporting the injection of automatic blood derivatives as PRP and i-PRF as safe and effective options in managing oral discomfort and lesions for patients who are unable to tolerate or do not respond well to traditional corticosteroid therapy. The findings from the Archana Shankar [30] and Sameeulla Shaik [42] studies provide further support for this conclusion by demonstrating no recurrence at one, three, and six-month follow-ups subsequent to the treatments administered. The study's results offer compelling evidence for the effectiveness of PRP and i-PRF in managing OLP, which presents a promising therapeutic option for individuals struggling with this condition. For patients who are unable to tolerate or do not respond well to traditional corticosteroid therapy, our findings may provide an alternative treatment approach.

When interpreting the findings of this review, several limitations need to be considered. Firstly, the included studies demonstrated moderate-to-severe heterogeneity that could be attributed to subjectivity in the measured indices. Based on the results of Begg's and Egger's tests, no significant of publication bias was detected in our meta-analysis. However, it is important to note that these tests do not guarantee the absence of publication bias, and other types of bias such as selective outcome reporting or language bias could still affect the results. Additionally, limited number of research included in the present meta-analysis may have reduced the power of these tests to detect publication bias. Due to the chronic nature and recurrence of OLP, short follow-up periods could lead to information bias and restrict the assessment of long-term clinical performance. Consequently, further research is required to determine the optimal dosage and frequency of Platelet concentrates injections for treating OLP and compare its long-term efficacy and potential adverse effects with those of other treatments [42]. In addition, longer-term studies are needed to determine the durability of the treatments and their potential side effects. Moreover, compared to some topical drugs, patients require timely follow-ups and demonstrate adherence to certain medical protocols. Cost-effectiveness analysis is therefore critical, given that the high cost of ACPs might impede access to some patients. Despite these limitations, our study supports the notion that ACPs represents a potentially effective and safe treatment option for OLP, particularly for patients who face increased risks of complications from corticosteroid therapy [43].

## Conclusions

Locally injected antigen-presenting cells, such as platelet-rich plasma or injectable platelet-rich fibrin, have demonstrated effectiveness in managing oral lichen planus. This suggests that they are a promising alternative to steroid therapy for OLP patients. In spite of the limitations of the present research, further study needed to determine long-term effectiveness and potential side effects of these therapies. Future studies should also investigate the mechanisms behind these therapies and establish standardized protocols for clinical efficacy.

### Supplementary Information

**Additional file 1.** 

**Additional file 2.** 

## Data Availability

The data used to support the findings of this study have been deposited in figshare at http://doi.org/10.6084/m9.figshare.22785206. Data of the study can be found in the Supplementary materials.
